# Hospitals

**Published:** 1876-06

**Authors:** 


### Hospitals.

### ST. LUKE'S HOSPITAL, CHICAGO.

1. *Extensive Syphilitic Disease of the Vault of the Cranium; Removal of two pieces of bone in their entire thickness, the larger piece measuring four by three inches; Recovery.*

(Under the care of JNO. E. OWENS, M.D.)

Mrs. K., aged 40 years, contracted syphilis five years ago. She has been under supervision during the past year for syphilitic disease of the larynx, and pain in the skull. In September, 1875, I found two soft doughy swellings, on the top of the head, each being the size of a silver quarter. Incisions having been made in each of these, the probe came in contact with denuded and roughened bone. The scalp was separated from the latter to the extent of several inches in every direction around these doughy swellings.

The diseased surface, which furnished a free discharge, had by means of the openings, proper drainage. The part was very offensive, requiring a liberal allowance of carbolized water. These two openings in the scalp subsequently enlarged, the one being 2 x 2 inches, two-thirds of which was to the right of the sagittal suture, and in close relation to the coronal suture; the other being 2 x ¾ inches.

At the earliest moment that the dead bone could be moved with a probe (Feb. 1, 1876), an incision was made across the scalp, from one of the openings above mentioned to the other, and the patient relieved of the offensive mass.

A cushion of soft cheesy matter covered the under surface of the roughened dead bone removed, thus affording some protection to the dura mater and the brain. The larger bone removed measured 4 x 3 inches; the other, 2 x 1. The opening in the skull, of course, corresponding in size and shape to the last bone, reached from a point $\frac{3}{4}$ inch on the left of the sagittal suture, $3\frac{1}{4}$ inches to its right side. The long diameter prolonged touched the left parietal protuberance and the outer canthus of right eye. Previous to the operation the patient had been a great sufferer in consequence of pain in the skull.

The medical treatment consisted for the most part of iodide of potassium and cod-liver oil. The larynx for months has been enlarged and stony. The patient could not speak above a whisper for a long time. The voice has not regained its natural tone, being more or less of a bleating quality. Sutures were used for the incised wounds, the openings in the scalp were filled with carbolized lint, a wad of oakum applied over all, and the part supported by moderate and diffused pressure. The frequency of the washings grew less as the discharge diminished and became less offensive.

No untoward symptoms supervening, the patient was permitted to get up at the expiration of 12 days. During this time she improved rapidly in general health, gaining both flesh and color.

Feb. 21st. Patient discharged.

April 1st. Wounds entirely healed.

Although the tissues that have filled in and closed the opening in the skull are very dense and firm, the pulsations in the brain are both readily felt and seen.

2. *Tracheotomy in Croup; Recovery.*

March 8th. I was called to see a little boy, aged six years, that was said to be dying. He "caught cold," Feb. 27th; began to breathe with difficulty, March 5th; and to have croupy cough, March 7th, with increasng dyspnœa. Paroxysms of difficulty of breathing supervened, exciting the alarm of the friends and rendering the case very distressing. Perspiration covered the forehead, upper lip and chin; the whole face wore a purple hue; pulse frequent; lungs normal; patient had a fair amount of strength.

No treatment had been used so far as could be learned, except the employment of the usual useless domestic remedies, so called. Deeming this a case where prompt relief to the breathing was demanded, I had the patient wrapped in a blanket and carried to the hospital, only a few doors distant, where, chloroform having been administered, the trachea was at once opened above the isthmus. A few detached pieces of false membrane were removed, and the tube introduced. The operation was attended by almost immediate relief. The first canula introduced, being too much curved, impinged against the anterior wall of the trachea, and had to be laid aside after several days. The bivalve canula is occasionally useful where there is difficulty in placing the instrument. It is, however, too likely to injure the lining of the trachea, and this danger increases with the frequency of the withdrawal of the inner tube. Whether one should arrest all hæmorrhage previous to opening the trachea, depends upon the urgency of the symptoms.

The operation is of but little use, unless some *competent* person is at hand to keep the tube clear. Dr. Hutchinson, the resident, saved this patient's life many times. An example: A paroxysm of dyspnœa came on; the inner tube was removed and cleansed in scalding water; dyspnœa increased; nothing in view obstructing the outer tube, and nothing could be wiped away; dyspnœa increasing to an alarming degree; inner tube replaced; no relief; both tubes removed; patient seemed to be almost dead; a drop or two of cold water into the wound excited a paroxysm of coughing, some inspissated mucus was expelled and the patient revived.

The medical treatment consisted of ferruginous tonics and an occasional anodyne. The patient found much comfort from the use of the steam atomizer, which played upon the wound.

March 15th. Patient able to speak, to blow and to whistle.

March 16th. Tube removed.

March 19th. Allowed to get up.

March 20th. Air does not pass from the wound.

March 29th. Patient discharged, well.

